# Targeting M2 Macrophages Alleviates Airway Inflammation and Remodeling in Asthmatic Mice *via* miR-378a-3p/GRB2 Pathway

**DOI:** 10.3389/fmolb.2021.717969

**Published:** 2021-09-13

**Authors:** Qiujie Wang, Luna Hong, Ming Chen, Jiangting Shi, Xiaoling Lin, Linjie Huang, Tiantian Tang, Yimin Guo, Xiaoqing Yuan, Shanping Jiang

**Affiliations:** ^1^ Division of Pulmonary and Critical Care Medicine, Sun Yat-sen Memorial Hospital, Sun Yat-sen University, Guangzhou, China; ^2^ Guangdong Provincial Key Laboratory of Malignant Tumor Epigenetics and Gene Regulation, Medical Research Center, Sun Yat-Sen Memorial Hospital, Sun Yat-sen University, Guangzhou, China; ^3^ Institute of Pulmonary Diseases, Sun Yat-sen University, Guangzhou, China; ^4^ Breast Tumor Center, Sun Yat-Sen Memorial Hospital, Sun Yat-Sen University, Guangzhou, China

**Keywords:** asthma, airway inflammation, airway remodeling, M2 macrophages, miR-378a-3p, GRB2

## Abstract

**Background:** Asthma is a complex respiratory disease characterized by airway inflammation and remodeling. MicroRNAs (miRNAs) mediate various cellular processes including macrophage polarization and play an important role in the pathogenesis of asthma. In present study, we aimed to screen miRNA profiling involved in macrophage polarization and investigate its possible functions and mechanisms.

**Methods:** An OVA-sensitized mouse model was established and 2-chloroadenosine (2-CA) was used to interfere with macrophages. The airway inflammation and remodeling were assessed. The identification and function of M2 alveolar macrophages were assessed by flow cytometry, RT-qPCR, arginase activity and co-culture experiment. Microarray screening was used to select miRNAs which were related to macrophage polarization and RNA interference (RNAi) technique was performed to confirm the function of the selected miRNA and its target gene.

**Results:** Alveolar macrophages of asthmatic mice showed significant M2 polarization. 2-CA alleviated airway inflammation and remodeling as well as M2 polarization. *In vitro*, IL-4-induced M2 macrophages promoted the proliferation of α-SMA-positive cells. And miRNA profiling showed a remarkable increased expression of miR-378a-3p in IL-4 induced M2 macrophages. Dual luciferase reporter assay confirmed growth factor receptor binding protein 2 (GRB2) was a target gene of miR-378a-3p. A miR-378a-3p inhibitor and knockdown of GRB2 repolarized alveolar macrophages from M1 to M2 phenotype.

**Conclusion:** Our findings suggest that miR-378a-3p/GRB2 pathway regulates the polarization of alveolar macrophages which acts as a potential therapeutic target for airway inflammation and remodeling in asthma.

## Introduction

Asthma is a chronic inflammatory airway disorder in which eosinophils, neutrophils, macrophages and CD4^+^ T cells migrate into the airways and release powerful mediators which lead to airway inflammation and remodeling (Anderson 2008; Holgate 2012; Fehrenbach et al., 2017). One of the most notable features of remodeling is airway smooth muscle cells hyperplasia, which strongly predicts airflow limitation and contributes to narrow airways (Brightling et al., 2012; James et al., 2012; Collaborators 2017).

Alveolar macrophages (AMs) have been recognized to play an important role in maintaining immunological homeostasis and host defense in the lungs. It has been reported that the depletion of AMs in mice with asthma attenuated airway inflammation and remodeling (Lee et al., 2015). 2-CA, a purine analog, might cause a competitive reduction in intracellular adenosine content once phagocytized by macrophages, thus reducing the viability of macrophages (Ohtani et al., 1982; Saito and Yamaguchi 1985). Kubota et al. reported that 2-CA would deplete the number of AMs in the bronchoalveolar lavage fluid of mice without any effect on neutrophil or lymphocyte counts (Kubota et al., 1999). Additionally, Hadjigol et al. found that the depletion of pulmonary macrophages by administration of 2-CA into the lungs suppressed airway hyperresponsiveness and reduced the expression of IL-13, TNF-α and IFN-γ (Hadjigol et al., 2020). Macrophages can be polarized into different phenotypes. M1 or ‘classically activated’ macrophages have high microbicidal and tumoricidal activity, as exemplified by the production of nitric oxide and pro-inflammatory cytokines such as IL-12 and TNF-α, while M2 or ‘alternatively activated’ macrophages have antiparasitic and tissue remodeling activity, characterized by the production of Arg-1, CD206, FIZZ1, Ym1, CCL17, and CCL24 (Liu and Yang 2013). It has been recognized that M2 macrophages were mainly activated by Th2 cytokines such as IL-4 and IL-13 (Gordon and Martinez 2010; Van Dyken and Locksley 2013). M2 macrophages have been known to facilitate the Th2 immune response and the secretion of chemokines and cytokines which regulate airway inflammation, tissue repair and airway remodeling in the lung (Murray and Wynn 2011; Yang et al., 2012; Byrne et al., 2015). Holtzman reported that the differentiation and accumulation of M2 macrophages might be a hallmark of allergic airway disease (Holtzman 2012).

miRNAs are noncoding small RNAs of 19–25 nucleotide in length which regulate characters of target sites, thus being responsible for diverse biological processes (Shenoy and Blelloch 2014). In our previous study, we have identified that miR-142-5p and miR-130a-3p regulated M2 macrophage polarization and promoted the expression of profibrogenic genes in chronic inflammation (Su et al., 2015). The expression of miRNAs in M2 alveolar macrophages and the mechanisms regarding its polarization require further investigation. In the present study, we aimed to screen miRNA profiling involved in macrophage polarization and investigate its possible functions and mechanisms. Through microarray screening, we identified the candidate miRNA responsible for macrophage polarization and confirmed its target gene.

## Materials and Methods

### Materials

OVA and 2-CA were purchased from Sigma (Missouri, United States). Recombinant murine IL-4 was sourced from Peprotech (New-Jersey, United States). Anti-CD80-PE, anti-CD206-BV650 and anti-CCR3-BV421 were purchased from Biolegend (California, United States). Antibodies for α-SMA and GRB2 were purchased from Abcam (Cambridge, England), F4/80 from Servicebio (Hangzhou, China), ECP-1 from Bioss (Beijing, China). The FISH kit, miRNA mimics, miRNA inhibitor and siRNAs were all purchased from GenePharma (Shanghai, China). The primers for PCR were synthesized by BGI (Beijing, China). The lipofectamine RNAiMax was purchased from Invitrogen (California, United States) while the X-tremeGENE HP DNA Transfection Reagent was purchased from Roche (Basel, Switzerland). The arginase assay kit was obtained from BioAssay (California, United States) and the Cell-Light Edu Apollo567 *In Vitro* Kit was purchased from Ribobio (Guangzhou, China). ELISA kits of IL-4 were from BD Biosciences (New-Jersey, United States) and the dual-Glo®Luciferase assay system was from Promega (Wisconsin, United States).

### Animals

Male BALB/c mice aged 6–8 weeks were obtained from the Beijing Vital River Laboratory Animal Technology Company and maintained in the Laboratory Animal Center of Sun Yat-sen University. All the experiments were performed in accordance with the protocol approved by the Institutional Animal Care and Use Committee at the Medical College of Sun Yat-sen University (Approval Number: 2017189).

### Establishment of Murine Model With Chronic Asthma

To produce further insights into the macrophage phenotype of asthma, an asthmatic model was established, and 2-CA was used to interfere with macrophages. Male BALB/c mice were randomly divided into three groups with eight mice in each group and treated as follows. 1) The control group were sham sensitized and boosted with normal saline. 2) In OVA group, mice were sensitized and challenged with OVA as previously described (Chen et al., 2011). They were sensitized by intraperitoneal injection (*i.p.*) of 10 μg of OVA emulsified in 1 mg of aluminium hydroxide in a total volume of 200 μl on Day-1 and Day-14. 7 days after the last sensitization (Day-21), mice were in atomization to 2.5% OVA aerosol for up to 30 min every 3 days for 8 weeks. 3) The 2-CA group was comprised of mice that were sensitized and challenged as in the OVA group described above and were additionally treated with 2 μM of 2-CA *via* intratracheal aerosolization using a compressor nebulizer (Dalian, China) before every subsequent three challenges of OVA. The mice were sacrificed 24 h after the final OVA challenge.

### BALF Analysis

Bronchoalveolar lavage fluid (BALF) was collected by lavage of lungs with 5 ml of precooled PBS *via* a tracheal cannula and centrifuged (3,000 rpm, 10 min, 4°C). The cells were used to detect the expression of CD80, CD206, and CCR3 by flow cytometry and the supernatants were analyzed for IL-4 by ELISA.

### Histology and Blood Analysis

The left lung lobes were harvested for histological analysis and the right lobes were snap-frozen in liquid nitrogen and stored at −80°C for RNA analysis. The blood was collected within heparinized tubes for flow cytometry analysis, while the plasma was kept at −80°C.

### Cell Culture and Differentiation

The murine alveolar macrophages (MH-S) purchased from ATCC (Cat. No.: CRL-2019) were maintained in RPMI 1640 containing 10% FBS supplemented with 1% penicillin/streptomycin and incubated at 37°C in a humidified incubator containing 5% CO_2_. To obtain M2 alveolar macrophages induced by IL-4 as previously described, MH-S cells were seeded into plastic 6-well plates at a concentration of 1 × 10^5^ cells/mL and incubated in serum-deprived medium for 12 h followed by treatment with IL-4 at 20 ng/ml for 48 h (Su et al., 2015). Then, the cells were harvested for further analysis.

### Culture of α-SMA-Positive Cells

Primary α-SMA-positive cells of mice were isolated by the tissue digestion method and purified by differential adhesion as previously described with slight modifications (Chen et al., 2016). Briefly, lungs were removed from 6-8-week-old male BALB/c mice and pulmonary tissues were scraped off. Bronchial tissues were cut into small pieces approximately 1 × 1 × 1 mm in size and incubated in RPMI 1640 containing 1 mg/ml collagenase I at 37°C for 4 h. Thereafter, the adherent cells within 30 min which were mainly airway fibroblasts were discarded and the suspended α-SMA-positive cells were pipetted into another culture flask. Successful isolation of α-SMA-positive cells was determined by immunofluorescence staining of α-SMA (Wang et al., 2020). α-SMA-positive cells were cultured in RPMI 1640 containing 10% FBS and passaged every 6–8 days, and cells from 4 to 9 passages were used for further experiments.

### Arginase Activity

To determine the polarization of alveolar macrophages, arginase assay was carried out following the manufacturer’s instructions (Ndolo et al., 2010). Total proteins were extracted from MH-S cells using ice-cold RIPA buffer containing 1 mM of PMSF. The supernatants of each sample were added into two separate wells of a 96-well microplate. 5X Substrate Buffer was added into one of the two wells, leaving the other one empty as a blank control. After incubation for 2 h at 37°C, urea reagent was added into all the wells while 5X Substrate Buffer was added into the blank control well. After further incubation for 1 h at room temperature, the optical density (OD) was measured by a microplate reader (Becan, Swissland) at 430 nm. Additionally, urea standard and ddH_2_O were used as standard and control, respectively. Arginase activity is calculated as (OD_sample_-OD_blank_)/(OD_standard_-OD_water_) × 10.4 (U/L).

### Co-Culture Experiment and EdU Incorporation Assay

For the co-culture experiment, transwell assay was carried out using a 24-well transwell apparatus with 0.4 μm pore size (Corning, New York, United States). α-SMA-positive cells were added into the lower chamber and MH-S cells were added into the upper chamber to explore the effect of M2 macrophages on α-SMA-positive cells. After co-culture for 48 h, proliferation of α-SMA-positive cells were determined using EdU kit according to the manufacturer’s instructions (Zhao et al., 2019). Cells were incubated with 50 μM 5-Ethynyl-2′-deoxyuridine (EdU) for 2 h followed by fixation with 4% paraformaldehyde and permeabilization with 0.5% Triton X-100. The incorporated EdU was visualized using Alexa Fluor 567 and the nuclear DNA was stained by Hoechst 33342. The images were obtained using a fluorescence microscope (Olympus, Tokyo, Japan).

### Flow Cytometry

Cells from BALF and MH-S cells were examined by flow cytometry. The amount of M1 macrophages (CD80^+^), M2 macrophages (CD206^+^) and eosinophils (CCR3^+^) were identified with specific antibodies and analyzed by multicolor flow cytometer (BD Celesta, New-Jersey, United States). The mean fluorescence intensity (MFI) was calculated by FlowJo software version 10 (Stanford, California, United States).

### Enzyme-Linked Immunosorbent Assay

The levels of IL-4 in BALF supernatants and plasma were determined by commercial ELISA kits following the manufacturer’s instructions. Briefly, samples were added into a 96-well microplate coated with capture antibody specific for murine IL-4. After incubation overnight at 4°C followed by three washes with washing buffer, detection antibody and streptavidin-peroxidase enzyme were added. After a further incubation for 1 h at room temperature and seven washes, substrate reagents were added, and the reaction was stopped by adding stop solution after 30 min. The intensity of the color was measured by a microplate reader (Tecan, Zurich, Switzerland) at 450 nm, which was subtracted by the absorbance at 570 nm. IL-4 protein levels were calculated with standard curves for each measurement.

### Histological Evaluation

The left lungs were perfused with PBS, fixed in 4% paraformaldehyde, embedded in paraffin and cut into 4 μm thick slices. Sections were stained with H&E, Periodic acid-Schiff (PAS) and Masson’s trichrome stain for evaluation of airway inflammation, mucus gland hyperplasia and collagen deposition, respectively. Semi-quantitative morphometric analysis was performed where the degrees of inflammation, mucus and collagen deposition were scored from 0 (absent) to 3 (severe), as modified from a previously described protocol (Stellari et al., 2015). The thickness of the airway wall was determined by morphometric analysis on transverse sections after α-SMA staining. F4/80 is a general marker for macrophages, and ECP-1 is a marker for eosinophils (Park et al., 2013). Sections were deparaffinized and rehydrated followed by microwave antigen retrieval in citric acid buffer (pH 6.0) and the blocking of endogenous peroxidase with 3% H_2_O_2_. Thereafter, sections were blocked with 5% BSA for 30 min at room temperature and incubated with the appropriate primary antibody overnight at 4°C in a humidified chamber. Staining was revealed followed by incubation with HRP-conjugated secondary antibody for 1 h at room temperature and the diaminobenzidine substrate kit for peroxidase. Counterstaining was performed using hematoxylin in 2 s.

### miRNA Microarray Analysis and Target Prediction

Total RNA from MH-S cells which were treated with or without 20 ng/ml of IL-4 for 48 h was isolated using TRIzol and purified with the RNeasy mini kit according to the manufacturer’s instructions. The samples were labelled using the miRCURY Hy3/Hy5 Power labelling kit (Exiqon) and hybridized on the miRCURY LNA Array (v.19.0, Exiqon). The slides were then scanned using the Axon GenePix 4000B microarray scanner. Scanned images were then imported into GenePix Pro 6.0 software (Axon) for grid alignment and data extraction. Replicated miRNAs were averaged and miRNAs with intensities ≥30 Units in all samples were chosen for calculation of the normalization factor. Expression data were normalized using median normalization. After normalization, differentially expressed miRNAs between two groups were identified through relative quantity, fold change (≥2.0) and *p*-value (<0.05). Finally, hierarchical clustering was performed to demonstrate distinguishable miRNA expression profiling among samples. The miRNA target prediction tools miRDB, TargetScan7.2 and DIANA-microT were used to identify target genes of candidate miRNAs. Kyoto Encyclopedia of Genes and Genomes (KEGG) analysis of the common target genes were further performed using the DAVID 6.8 website (https://david.ncifcrf.gov/home.jsp).

### Quantitative Real-Time PCR

Total RNA from MH-S cells or lungs was extracted using TRIzol according to the manufacturer’s instructions. RNA was converted to cDNA with PrimeScript Master Mix for mRNA detection and with Mir-X miRNA First-Strand Synthesis Kit for miRNA evaluation. RT-qPCR was performed using the TB Green Premix Ex Taq II in a LightCycler 480 instrument (Roche, Basel, Switzerland). The fold-change of the transcript mRNA or miRNA was analyzed using the 2^−ΔΔC^T method. Expression of mRNA was normalized to that of β-ACTIN, and mature miRNAs were calculated using U6 as an internal control. The gene-specific primer sequences used for mRNA are listed in Table 1 and the primers for miRNAs are listed in Table 2.

**TABLE 1:** The mRNA-specific primer sequences.

mRNA name	Forward (5′-3′)	Reverse (5′-3′)
IL-6	CCT​CTG​GTC​TTC​TGG​AGT​ACC	ACT​CCT​TCT​GTG​ACT​CCA​GC
IL-10	TGC​CTG​CTC​TTA​CTG​ACT​GG	CTC​TAG​GAG​CAT​GTG​GCT​CTG
IL-12	CCC​TTG​CCC​TCC​TAA​ACC​A	CTA​AGA​CAC​CTG​GCA​GGT​CCA
CCL5	ATA​TGG​CTC​GGA​CAC​CAC​TC	ACT​TGG​CGG​TTC​CTT​CGA​G
CCL24	CTG​TCT​GTC​TGT​CCA​TCT​CTG​G	GCT​GCT​GTT​GAA​ATC​CTC​CGT​T
iNOS	TTC​ACC​CAG​TTG​TGC​ATC​GAC​CTA	TCC​ATG​GTC​ACC​TCC​AAC​ACA​AGA
FIZZ1	TGC​TGG​GAT​GAC​TGC​TAC​TG	AGC​TGG​GTT​CTC​CAC​CTC​TT
Arg1	TTG​GCT​TGC​TTC​GGA​ACT​CA	TTC​ATG​TGG​CGC​ATT​CAC​AG
GRB2	AAC​ATC​CGT​GTC​CAG​GAA​CC	AAG​TCT​CCT​CTG​CGA​AAG​CC
β-ACTIN	GAT​CAG​CAA​GCA​GGA​GTA​CGA	CAG​CTC​AGT​AAC​AGT​CCG​C

**TABLE 2:** The miRNA-specific primer sequences.

miRNA name	Sequences (5′-3′)
Universal primer	GTGCGTGTCGTGGAGTCG
miR-431-5p	AGGTGTCTTGCAGGCCGT
miR-677-3p	AAGCCAGATGCCGTTCCT
Let-7i-3p	GGC​TCT​GCG​CAA​GCT​ACT​G
miR-3093-5p	CGC​GGA​GCT​CAC​ACT​AAA​A
miR-378a-3p	GGG​CAC​TGG​ACT​TGG​AGT​C
miR-370-5p	GGG​ACA​GGT​CAC​GTC​TCT​GC
U6-Forward	GCT​TCG​GCA​GCA​CAT​ATA​CTA​AAA​T
U6-Reverse	CGC​TTC​ACG​AAT​TTG​CGT​GTC​AT

### Fluorescence *In situ* Hybridization

Fluorescence *in situ* hybridization was performed on paraffin-embedded sections of lungs according to the manufacturer’s instructions. First, the sections were deparaffinized and rehydrated. After rinsing with PBS, the slides were digested with protease K for 20 min at 37°C and then dehydrated by ethanol. The sections were prehybridized in denaturation solution for 8 min at 78°C and dehydrated by ethanol again. Then the sections were hybridized with 20 ng/μl Cy3-labelled LNA miR-378a-3p probe overnight at 37°C. After post-hybridization washes, the nuclei were counterstained with DAPI and the fluorescence images were captured by a confocal microscope (Olympus, Tokyo, Japan).

### Transfection of miRNAs or siRNAs

To determine the effect of miR-378a-3p or GRB2 on polarization of alveolar macrophages or the influence of miR-378a-3p on GRB2, we used RNAi to silence miR-378a-3p or GRB2. MH-S cells were seeded into 6-well plates at a density of 1 × 10^5^ cells/ml, and transfection was performed when cells were at 60∼70% confluence. 100 pmol of miR-378a-3p mimics, miR-378a-3p inhibitor, siRNA specific to GRB2 (si-GRB2) or negative control siRNA (si-NC) were mixed with 250 μl Opti-MEM, and 5 μl Lipofectamine RNAiMax was mixed with 250 μl Opti-MEM for 5 min. Then the RNA complexes and liposome complexes were mixed together, incubated for 20 min and transfected into cells. The cells were harvested 48 h later for further analysis. Sequences of miR-378a-3p inhibitor and si-GRB2 are provided in Table 3.

**TABLE 3:** Sequences of the specific miRNA and siRNA.

Sequence name	Forward (5′-3′)	Reverse (5′-3′)
miR-378a-3p mimic	ACU​GGA​CUU​GGA​GUC​AGA​AGG	UUC​UGA​CUC​CAA​GUC​CAG​UUU
miRNA mimics NC	UUC​UCC​GAA​CGU​GUC​ACG​UTT	ACG​UGA​CAC​GUU​CGG​AGA​ATT
miR-378a-3p inhibitor	CCU​UCU​GAC​UCC​AAG​UCC​AGU	—
miRNA inhibitor NC	CAG​UAC​UUU​UGU​GUA​GUA​CAA	—
si-GRB2	GGA​ACC​AGC​AGA​UAU​UCU​UTT	AAG​AAU​AUC​UGC​UGG​UUC​CTT
siRNA NC	UUC​UCC​GAA​CGU​GUC​ACG​UTT	ACG​UGA​CAC​GUU​CGG​AGA​ATT

### Western Blot

Protein lysates extracted from lung tissues or MH-S cells which were transfected with miR-378a-3p mimics, miR-378a-3p inhibitor, or si-GRB2 were centrifuged at 16,000 *g* for 20 min at 4°C and quantified using the BCA method. 20 μg of proteins were separated by 10% SDS-PAGE and transferred onto PVDF membranes. After blocking, blots were probed with primary antibodies against GRB2 or β-tubulin at 4°C overnight followed by incubation with HRP-conjugated secondary antibody for 1 h at room temperature. Protein bands were detected by enhanced chemiluminescence assay and the blots were visualized by Minichemi Imaging System (Beijing, China). Densitometric quantitation of band intensity was carried out with ImageJ software version 5.0 (NIH, Maryland, United States).

### Dual Luciferase Reporter Assay

To identify the binding site between miR-378a-3p and GRB2, the 3′ untranslated region (UTR) of GRB2 including wild type (WT) or mutant type (MUT) of the binding site was synthesized and cloned into the SV40-firefly_Luciferase-MCS vectors (Genechem, Shanghai, China) and 293T cells were used for reporter assays. 293T cells (5 × 10^4^ cells per well) were seeded into 24-well plates and transfected with miR-378a-3p mimics or negative control (20 pmol) for 24 h when the cells reached 60–70% confluence. Then cells were co-transfected with WT or MUT GRB2 3′ UTR vector and the control vector coding for Renilla luciferase using X-tremeGENE HP DNA transfection reagent for 48 h. Cells were harvested using 300 μl of Passive Lysis Buffer and luciferase activities were measured by a microplate reader (Zeng et al., 2019). The firefly luciferase activity was normalized to that of Renilla luciferase. Sequences of miR-378a-3p mimics and negative control are provided in Table 3.

### Statistical Analysis

The quantitative data were presented as the mean ± SEM. For quantification analysis, each experiment was performed at least three times. Statistical analysis was conducted using GraphPad Prism software version 5.0 (GraphPad, California, United States) and SPSS software version 20.0 (SPSS, Chicago, United States). Analysis between two groups was performed using Student’s t-test and multiple comparisons of continuous variables were analyzed using one-way analysis of variance. Differences were considered statistically significant when the *p*-value was less than 0.05.

## Results

### Airway Inflammation and Remodeling Along With M2 Macrophage Polarization in OVA-Sensitized Mice With Chronic Asthma

**FIGURE 1:**
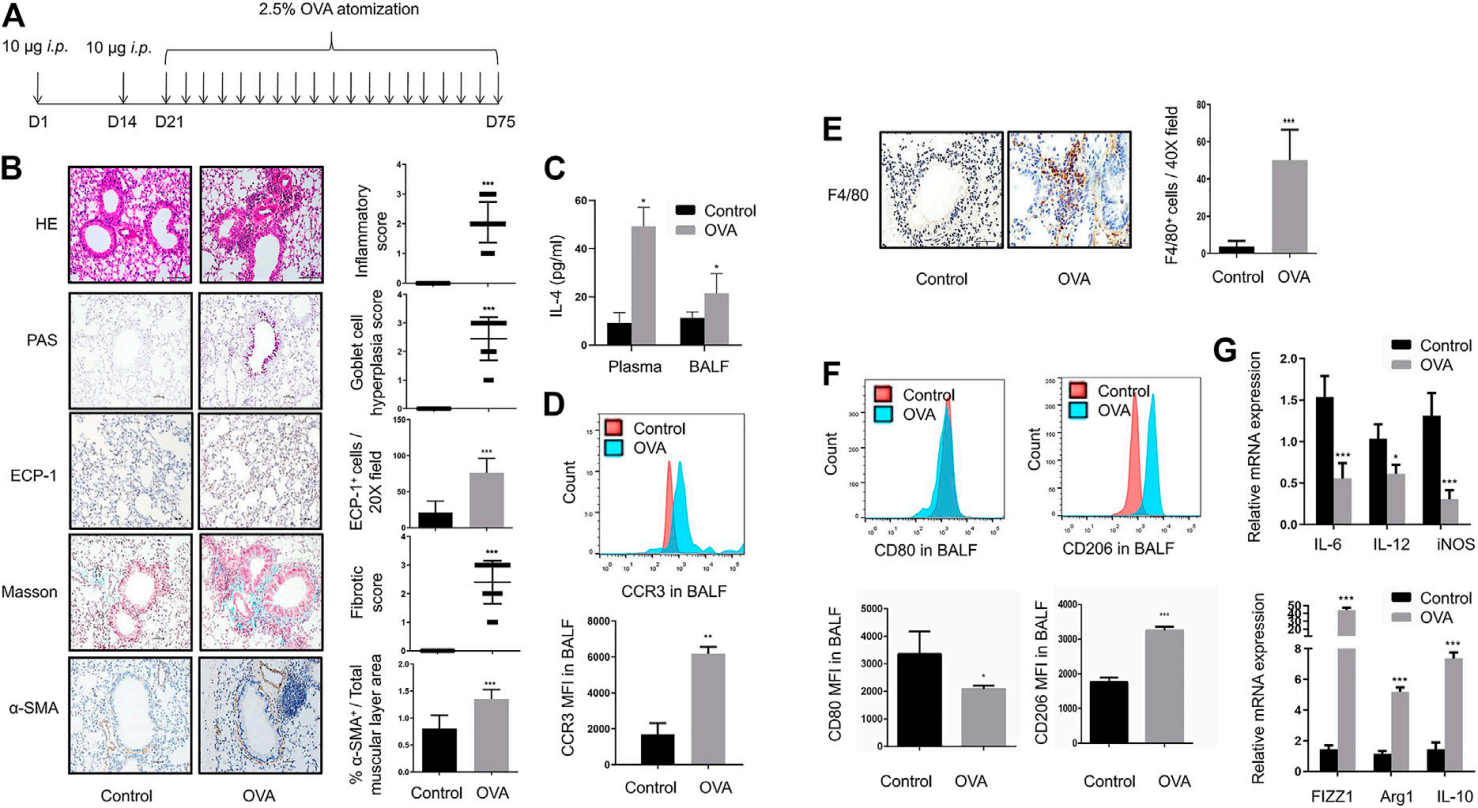
Airway inflammation and remodeling along with M2 macrophage polarization in OVA-sensitized mice with chronic asthma **(A)**. Timeline of establishment of the chronic asthma model which was sensitized and challenged with OVA for 8 weeks **(B)**. Representative images of HE, PAS, Masson, ECP-1, and α-SMA immunohistochemistry staining of lung sections from control and OVA-sensitized mice as shown at a magnification of 20X. Scale bar: 50 μm. Bar graph represent morphometric semi-quantitative analysis of histopathological data from HE, PAS, and Masson staining. Quantification of ECP-1^+^ cells per 20X field and percentage of α-SMA^+^/total muscular layer area **(C)**. IL-4 levels in plasma and BALF from control and OVA-sensitized mice assessed by ELISA **(D)**. Expression of CCR3 in BALF from control and OVA-sensitized mice as determined by flow cytometry analysis. The representative histograms and quantitation of the MFI are shown **(E)**. Representative images of F4/80 immunohistochemistry staining of lung sections from control and OVA-sensitized mice as shown at a magnification of 40X. Scale bar: 50 μm. Quantification of F4/80^+^ cells per 40X field **(F)**. Expression of CD80 and CD206 in alveolar macro-phages of BALF from control and OVA-sensitized mice as determined by flow cytometry analysis. The representative histograms and quantitation of the MFI are shown **(G)**. Expression of IL-6, IL-12, iNOS, FIZZ1, Arg1, and IL-10 in lungs from control and OVA-sensitized mice evaluated by RT-qPCR. (*n* = 8, *p < 0.05, **p < 0.01, ***p < 0.001 vs. control mice).

In the present study, we have developed an OVA-sensitized mouse model (Figure 1A). In asthmatic mice, HE staining revealed inflammatory cell infiltration; a large number of PAS^+^ goblet cells were detected by PAS staining; immunohistochemical staining of ECP-1 showed a significant increase of eosinophilic infiltration; the total amount of collagen was significantly increased as defined by Masson staining; and marked airway smooth muscle cell hyperplasia and hypertrophy have been identified by α-SMA immunohistochemical staining of the lungs (Figure 1B). IL-4 levels of plasma and BALF in OVA-challenged mice were significantly increased (Figure 1C). The results of flow cytometry analysis showed that the MFI of eosinophils in BALF was increased in OVA-challenged mice (Figure 1D). These data showed the airway inflammation and remodeling in OVA-sensitized mice.

A large number of F4/80^+^ macrophages were detected around asthmatic airways in the lungs of asthmatic mice (Figure 1E). With OVA challenges, a significant downregulation of CD80^+^ M1 macrophages and upregulation of CD206^+^ M2 macrophages were detected in the BALF of asthmatic mice (Figure 1F). In addition, OVA challenge resulted in a significant increase in M2 markers such as FIZZ1, Arg1, and IL-10, with a decrease in M1 markers such as IL-6, IL-12, and iNOS (Figure 1G). These results indicated strong M2 macrophage infiltration in the BALF and lungs of asthmatic mice.

### Inhibition of M2 Macrophage Polarization by 2-CA is Accompanied by Alleviation of Airway Inflammation and Remodeling

**FIGURE 2:**
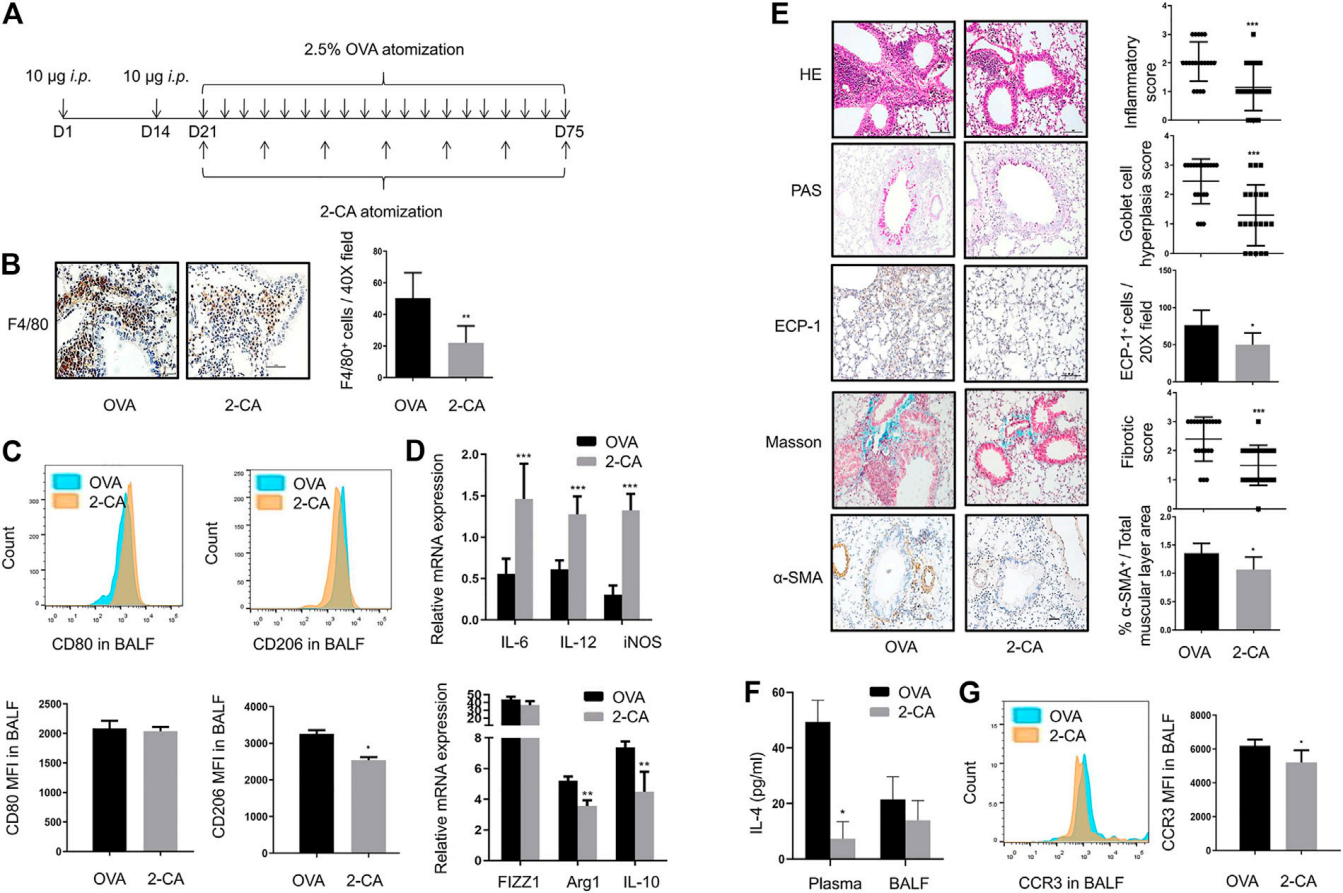
Inhibition of M2 macrophage polarization by 2-CA is accompanied by alleviation of airway inflammation and remodeling **(A)**. Timeline of mice with chronic asthma treated with 2-CA before every subsequent three challenges of OVA **(B)**. Representative images of F4/80 immunohistochemistry staining of lung sections from OVA-sensitized and 2-CA-treated mice as shown at a magnification of 40X. Scale bar: 50 μm. Quantification of F4/80^+^ cells per 40X field **(C)**. Expression of CD80 and CD206 in alveolar macrophages of BALF from OVA-sensitized and 2-CA-treated mice as determined by flow cytometry analysis. The representative histograms and quantitation of the MFI are shown **(D)**. Expression of IL-6, IL-12, iNOS, FIZZ1, Arg1, and IL-10 in lungs from OVA-sensitized and 2-CA-treated mice evaluated by RT-qPCR **(E)**. Representative images of HE, PAS, Masson, ECP-1, and α-SMA immunohistochemistry staining of lung sections from OVA-sensitized and 2-CA-treated mice as shown at a magnification of 20X. Scale bar: 50 μm. Bar graph represent morphometric semi-quantitative analysis of histopathological data from HE, PAS and Masson staining. Quantification of ECP-1^+^ cells per 20X field and percentage of α-SMA^+^/total muscular layer area **(F)**. IL-4 levels in plasma and BALF from OVA-sensitized and 2-CA-treated mice assessed by ELISA **(G)**. Expression of CCR3 in BALF from OVA-sensitized and 2-CA-treated mice as determined by flow cytometry analysis. The representative histograms and quantitation of the MFI are shown. (*n* = 8, *p < 0.05, **p < 0.01, ***p < 0.001 vs. asthmatic mice).

The timeline in which 2-CA was used to reduce AMs was depicted in Figure 2A. Treatment with 2-CA effectively reduced the number of F4/80^+^ macrophages in asthmatic mice (Figure 2B). With 2-CA management, the downregulation of CD80^+^ M1 macrophages and upregulation of CD206^+^ M2 macrophages in BALF of asthmatic mice could be reversed (Figure 2C). In addition, 2-CA challenge within asthmatic mice resulted in a decrease in M2 markers such as FIZZ1, Arg1, and IL-10 and an increase in M1 markers such as IL-6, IL-12, and iNOS, which suggested that 2-CA could inhibit M2 polarization in asthmatic mice (Figure 2D). Meanwhile, the inflammatory cell infiltration, goblet cell hyperplasia, eosinophilic infiltration, subepithelial fibrosis, and smooth muscle cell hyperplasia and hypertrophy in the lungs of asthmatic mice could be alleviated by 2-CA treatment (Figure 2E). Consistent with this finding, the increased IL-4 levels of plasma and BALF and MFI of eosinophils in BALF in OVA-challenged mice were reduced by 2-CA (Figures 2F,G). The results suggested that 2-CA could attenuate airway inflammation and remodeling in parallel with the reduction of M2 macrophages in asthmatic mice.

### IL-4-Induced M2 Macrophages Promote the Proliferation of α-SMA-positive Cells *in vitro*

**FIGURE 3:**
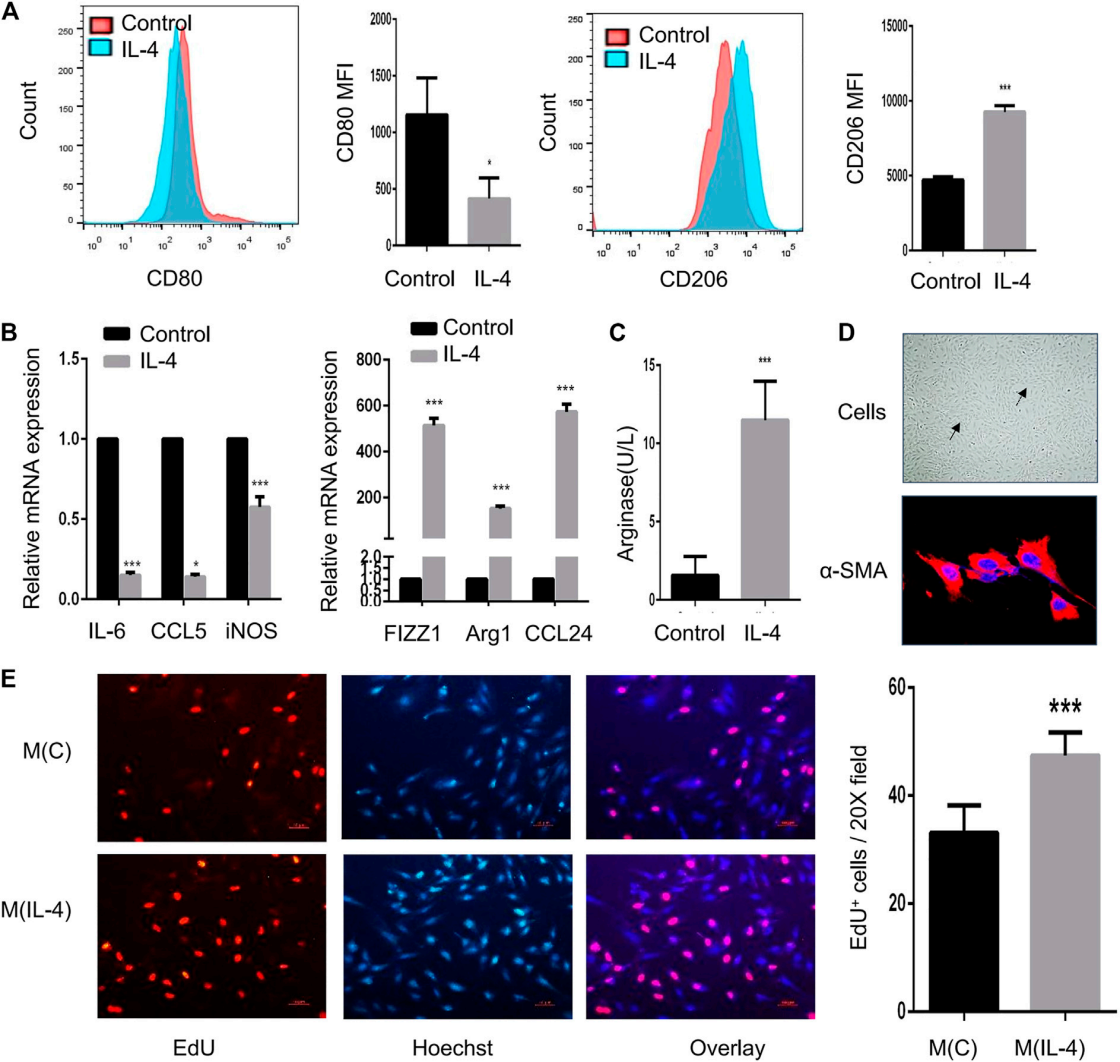
IL-4-induced M2 macrophages promote the proliferation of α-SMA-positive cells *in vitro*. MH-S cells were treated for 48 h with 20 ng/ml IL-4, and the identification and function of IL-4-induced M2 macrophages were analyzed **(A–C) (A)**. Expression of CD80 and CD206 in IL-4-induced M2 macrophages evaluated by flow cytometry analysis. The representative histograms and quantitation of the MFI are shown **(B)**. Expression of IL-6, CCL5, iNOS, FIZZ1, Arg1, and CCL24 in IL-4-induced M2 macrophages evaluated by RT-qPCR **(C)**. Arginase in IL-4-induced M2 macrophages measured by the arginase assay **(D)**. Representative image of α-SMA-positive cells under light microscope and immunofluorescent staining of α-SMA as shown at a magnification of 40X. Scale bar: 50 μm. The solid arrow points to the hill and the dotted arrow points to the valley **(E)**. MH-S cells were stimulated with 20 ng/ml IL-4 for 48 h and co-cultured with primary α-SMA-positive cells for another 48 h. M(C) means MH-S cells treated without IL-4 and M(IL-4) means with IL-4. The proliferation of α-SMA-positive cells detected by EdU incorporation assay as shown at a magnification of 20X. Scale bar: 10 μm. Quantitation of percentage of EdU^+^ cells per 20X field. (*p < 0.05, ***p < 0.001 vs. control).

In addition to *in vivo* experiment, macrophage polarization also affects the function of airway cells *in vitro*. After IL-4 induction, MH-S cells displayed a highly enhanced expression of CD206 and a markedly reduced expression of CD80 by flow cytometry (Figure 3A). Also, the mRNA expression of FIZZ1, Arg1, and CCL24 were largely increased (*p* < 0.05) while IL-6, CCL5, and iNOS were significantly reduced (Figure 3B). Besides, the expression of arginase was significantly increased (Figure 3C). To investigate the effect of M2 macrophages on α-SMA-positive cells, MH-S cells were stimulated with IL-4 and then co-cultured with α-SMA-positive cells to evaluate the proliferative ability of α-SMA-positive cells. As shown in Figure 3D, primary α-SMA-positive cells were identified by the typical “hill and valley” growth pattern and immunofluorescent staining of α-SMA. The proliferative ability of α-SMA-positive cells was strongly enhanced when co-cultured with IL-4-induced M2 macrophages (Figure 3E).

### MiR-378a-3p is Upregulated in IL-4-Induced M2 Macrophages

**FIGURE 4:**
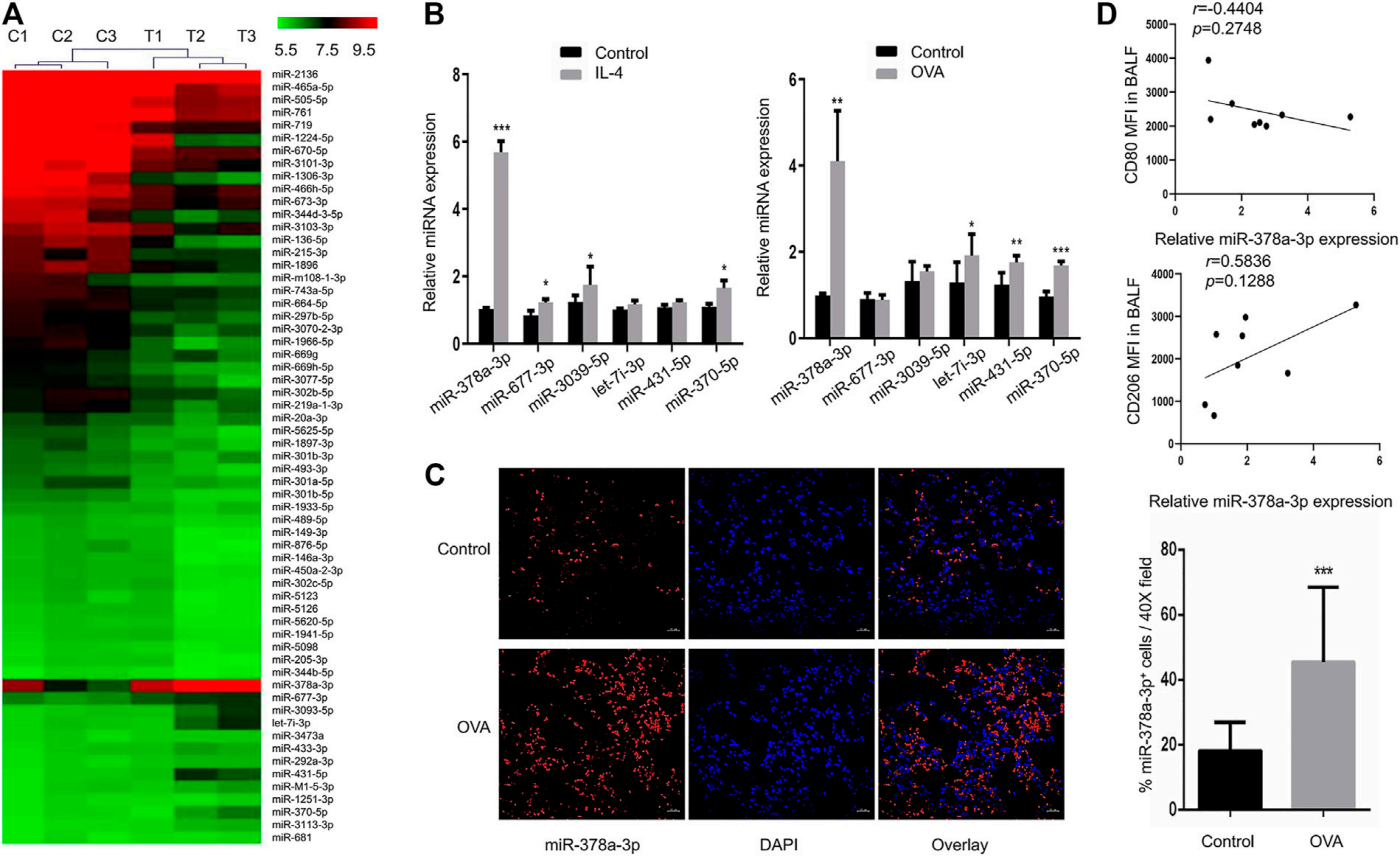
MiR-378a-3p is upregulated in IL-4-induced M2 macrophages **(A)**. Differential expression levels of miRNAs (fold changes ≥2 and *p*-value < 0.05) in MH-S cells treated with or without 20 ng/ml IL-4 for 48 h were presented in a heatmap. The mean fluorescence intensity was calculated as the average for three replicates **(B)**. Expression levels of the candidate miRNAs in MH-S cells following 20 ng/ml IL-4 for 48 h treatment and in the lungs of control and OVA-sensitized mice as determined by RT-qPCR **(C)**. Representative images of FISH for miR-378a-3p in the lung tissues of control and OVA-sensitized mice as shown at a magnification of 40X. Scale bar: 20 μm. Quantitation of percentage of miR-378a-3p^+^ cells per 40X field **(D)**. Scatter plots of CD80, CD206 MFI in BALF and relative miR-378a-3p expression from control and OVA-sensitized mice. Individual data points are shown with *p* value and linear regression. (*n* = 8, *p < 0.05, **p < 0.01, ***p < 0.001 vs. control).

To explore the important roles of miRNAs in macrophage polarization, we identified differentially expressed miRNAs in murine alveolar macrophages (MH-S) exposed to IL-4 *via* miRNA microarray analysis. The results showed that 13 miRNAs were upregulated, and 49 miRNAs were down-regulated between groups (Figure 4A). We further determined the expression of a subset of miRNAs to validate the microarray results by RT-qPCR (Figure 4B). Among these miRNAs, miR-677-3p, miR-3093-5p, miR-378a-3p, and miR-370a-5p were found to be significantly upregulated. The same subset of miRNAs was detected in the lungs of OVA-challenged mice by RT-qPCR (Figure 4B). Notably, miR-378a-3p was the most upregulated miRNA both in IL-4-induced M2 macrophages and the lungs of mice with chronic asthma, which might be involved in M2 polarization of macrophages (Vats et al., 2006; Eichner et al., 2010). In accordance with the results, FISH analysis also showed an increase of miR-378a-3p expression in the lungs of asthmatic mice (Figure 4C). Besides, the correlation coefficient between CD80 MFI in BALF and relative miR-378a-3p expression was negative while the correlation coefficient between CD206 MFI in BALF and relative miR-378a-3p expression was positive, though both of them didn’t achieve statistical significance (Figure 4D).

### MiR-378a-3p Directly Targets GRB2 in Murine Alveolar Macrophages

**FIGURE 5:**
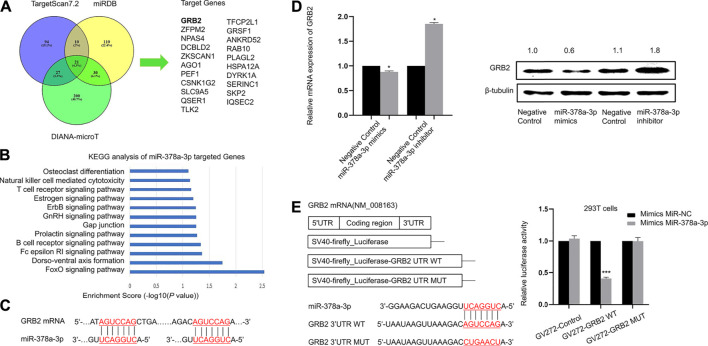
miR-378a-3p directly targets GRB2 in murine alveolar macrophages **(A)**. Target gene prediction of miR-378a-3p with three bioinformatics tools and the results are shown in the Venn diagram **(B)**. KEGG pathway analysis of target genes of miR-378a-3p **(C)**. The binding sites between miR-378a-3p and target gene GRB2 **(D)**. RT-qPCR and Western blotting assay of GRB2 expression in MH-S cells treated with miR-378a-3p mimics and miR-378a-3p inhibitor or control **(E)**. Diagram of the wild-type and a mutated-type of binding site between miR-378a-3p and GRB2 and luciferase reporter assays for 293T cells transfected with GV272 vectors carrying GRB2-3′ UTR *versus* GRB2-MUT-3′ UTR in the absence or presence of miR-378a-3p mimics. (*p < 0.05, **p < 0.01, ***p < 0.001 vs. control).

We used different target-prediction algorithms including miRDB, TargetScan7.2 and DIANA-microT to screen for mRNA targets of miR-378a-3p. 21 target genes were predicted by all three databases (Figure 5A). Among these target genes, KEGG_PATHWAY analysis suggested that GRB2 might participate in osteoclast differentiation and T cell receptor signaling pathway which was associated with macrophage polarization. Therefore we hypothesized that GRB2 might participate in alveolar macrophage differentiation since osteoclasts acted as a type of macrophage (Figure 5B). Firstly, we found two identical seed-matching sites exist between the 3′-UTR of GRB2 and miR-378a-3p (Figure 5C). And there is no target site at the 3′UTR of GRB2 for miRNAs identified in microarray analysis besides miR-378a-3p. To validate whether GRB2 was a target gene of miR-378a-3p, we further carried out gain and loss of function analysis as well as dual luciferase reporter assay. The mRNA and protein expression of GRB2 were significantly decreased after MH-S cells were transfected with miR-378a-3p mimics and increased after transfected with miR-378a-3p inhibitor, implying that the reduced transcription and translation of GRB2 caused by miR-378a-3p was reversed by miR-378a-3p inhibitor (Figure 5D). Furthermore, the results of dual luciferase reporter assay showed that the luciferase activity decreased obviously in cells co-transfected with the wild-type binding site vector in the presence of miR-378a-3p. On the contrary, cells containing the mutated binding sites vector did not show such suppression (Figure 5E). These data confirmed that GRB2 was a specific target of miR-378a-3p.

### MiR-378a-3p Contributes to Alveolar Macrophage Polarization by Targeting GRB2

**FIGURE 6:**
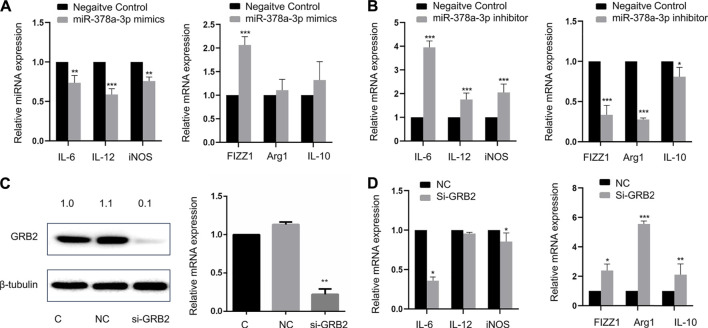
**(A)** MiR-378a-3p contributes to alveolar macrophage polarization by targeting GRB2 Expression of IL-6, IL-12, iNOS, FIZZ1, Arg1, and IL-10 in MH-S cells treated with 100 pmol miR-378a-3p mimics or negative control for 48 h valuated by RT-qPCR **(B)**. Expression of IL-6, IL-12, iNOS, FIZZ1, Arg1, and IL-10 in MH-S cells treated with 100 pmol miR-378a-3p inhibitor or negative control for 48 h evaluated by RT-qPCR **(C)**. GRB2 mRNA and protein in MH-S cells treated with 100 pmol si-GRB2 for 48 h measured by RT-qPCR and Western Blot **(D)**. Expression of IL-6, IL-12, iNOS, FIZZ1, Arg1, and IL-10 in MH-S cells treated with 100 pmol si-GRB2 for 48 h evaluated by RT-qPCR. (*p < 0.05, **p < 0.01, ***p < 0.001 vs. control).

As showed in Figure 6A, M1 markers such as IL-6, IL-12, and iNOS were downregulated while M2 markers such as FIZZ1, Arg1, and IL-10 were upregulated in alveolar macrophages transfected with miR-378a-3p mimics. When alveolar macrophages were transfected with miR-378a-3p inhibitor, M1 markers were upregulated while M2 markers were downregulated (Figure 6B), suggesting that miR-378a-3p might promote M2 polarization of alveolar macrophages. The function of GRB2 in alveolar macrophage polarization was evaluated by silencing GRB2 expression with RNAi. As shown in Figure 6C, the mRNA and protein expression of GRB2 was significantly repressed after si-GRB2 silencing (*p* < 0.05). Silencing of GRB2 also resulted in a downregulation of IL-6, IL-12, and iNOS mRNA with an upregulation FIZZ1, Arg1, and IL-10 mRNA in alveolar macrophages (Figure 6D). These data suggested that miR-378a-3p might contribute to M2 polarization of alveolar macrophages *via* the direct downregulation of GRB2.

## Discussion

Since M2/M1 imbalance has been a recognized pathology in asthma, studies have been conducted to explore the therapeutic potential of repolarizing M2 to M1 macrophages. In this study, we found that M2 macrophages were the predominant phenotype in the lungs and BALF of mice with chronic asthma induced by OVA. We also observed that the predominant M2 phenotype could be repolarized to the M1 phenotype by 2-CA in mice with chronic asthma, which was accompanied by the alleviation of airway inflammation and remodeling. These data suggested that 2-CA could reduce airway inflammation and remodeling in asthmatic mice by shifting macrophages from M2 to M1.

Recently, miRNAs have been found to affect the M1/M2 macrophage polarization (Self-Fordham et al., 2017; Li et al., 2018). For example, miRNA-9, miRNA-27, miRNA-155 and miRNA-125b could promote M1 polarization while miRNA-21, miRNA-223, miRNA-34, let-7c, miRNA-146a, and miRNA-511 could promote M2 polarization in various tissues (Feketea et al., 2019). However, the role of miRNAs in M2 alveolar macrophage polarization still needs more explorations. A continuous cell line of murine alveolar macrophages, designated MH-S, has been established and been used in our experiments. In our study, we confirmed that MH-S cells displayed a M2 phenotype after IL-4 induction. Then, differentially expressed miRNAs in M2 MH-S cells were screened, and miR-378a-3p was the most significantly increased miRNA in M2 MH-S cells and the lungs of mice with chronic asthma. miR-378a-3p is encoded in an intronic region of PPARGC1b, a protein associated with M2 polarization of macrophages (Vats et al., 2006; Eichner et al., 2010). Li et al. reported that miR-378 was increased in the peripheral blood and lungs of asthmatic children (Li et al., 2019). Rückerl et al. reported that miR-378-3p was significantly upregulated by IL-4 in peritoneal macrophages and was involved in IL-4-driven macrophage proliferation in mice implanted with *Brugia malayi* nematodes (Ruckerl et al., 2012), which didn’t mention the role of downstream gene targets. In our study, we found that miR-378a-3p inhibitor promoted a shift from the M2 to M1 phenotype of alveolar macrophages, which meant that miR-378a-3p might participate in M2 polarization of alveolar macrophages.

With online target prediction tools, we have identified GRB2 as a target of miR-378a-3p, which have been verified by gain and loss of function analysis as well as luciferase reporter assay. GRB2 is a receptor-bound protein in various cells which link signaling events initiated by tyrosine kinases to downstream pathways. It can directly bind to activated EGF receptor phosphorylated tyrosine, participate in EGF receptor mediated signal transduction, and indirectly participate in insulin receptor-mediated signal transduction by binding with SHC phosphorylated tyrosine. GRB2 can combine with SHC and SOS at the same time to form SHC-GRB2-SOS complex and activate SOS. The activated SOS can combine with Ras protein on plasma membrane, and activate it to cause signal cascade reaction. (Rao 1996). It is reported that reducing GRB2 expression by shRNA or by gene targeting slowed down the osteoclast-like differentiation of cells (Levy-Apter et al., 2014). CD206 activation mediated GRB2 recruitment to initiate phagocytosis signaling in human monocyte-derived macrophages (Rajaram et al., 2017). Several studies have shown the important effects of GRB2 in eosinophils, mast cells and T cells in allergic diseases (Ben Baruch-Morgenstern et al., 2014; Massoud et al., 2016; Lin et al., 2018). Xu et al. reported that miR-378a-3p sensitized ovarian cancer cells to cisplatin through targeting MAPK1/GRB2 (Xu et al., 2018). Yu et al. suggested that LMV induced osteogenic differentiation of BMSCs through miR-378a-3p/Grb2 pathway to improve bone mineral density and mechanical properties (Yu et al., 2020). However, whether GRB2 participated in alveolar macrophage polarization is still unknown. We demonstrated that the knockdown of GRB2 resulted in repolarization of alveolar macrophages from the M1 to M2 phenotype. Taken together, these findings indicated that the miR-378a-3p/GRB2 axis might act as a mediator of M2 polarization of alveolar macrophages. However, whether other miRNAs or target genes participate in functions of macrophage deserves further experiments.

In conclusion, our study has provided evidences that miR-378a-3p is upregulated in M2 alveolar macrophages which aggravate airway inflammation and remodeling in asthmatic mice. Persistent elevation of miR-378a-3p levels in alveolar macrophages promotes the switch towards the M2 phenotype by suppressing the expression of GRB2. Our results provide the possibility that the inhibition of miR-378a-3p may serve as a promising therapeutic strategy for chronic asthma which deserves further *in vivo* experiments.

## Biosecurity Statement

All the experiments were performed in Guangdong Provincial Key Laboratory of Malignant Tumor Epigenetics and Gene Regulation, Medical Research Center, Sun Yat-Sen Memorial Hospital and in the Laboratory Animal Center of Sun Yat-sen University, which strictly adhered to standard biosecurity and institutional safety procedures.

## Data Availability

The data presented in the study are deposited in the GEO repository, accession number GSE143740.

## Abbreviations

AMs, alveolar macrophages; BALF, bronchoalveolar lavage fluid; GRB2, growth factor receptor binding protein 2; miRNAs, microRNAs; MH-S, murine alveolar macrophages; M1, classically activated macrophages; M2, alternatively activated macrophages; OVA, ovalbumin; 2-CA, 2-chloroadenosine.
