# Supplementary material for: Targeting M2 Macrophages Alleviates Airway Inflammation and Remodeling in Asthmatic Mice via miR-378a-3p/GRB2 Pathway
Source: Front Mol Biosci. 2021 Sep 13;8:717969. doi: 10.3389/fmolb.2021.717969 (PMC8473897; doi:10.3389/fmolb.2021.717969)
Supplement: Supplementary file 2 [file DataSheet4.doc]

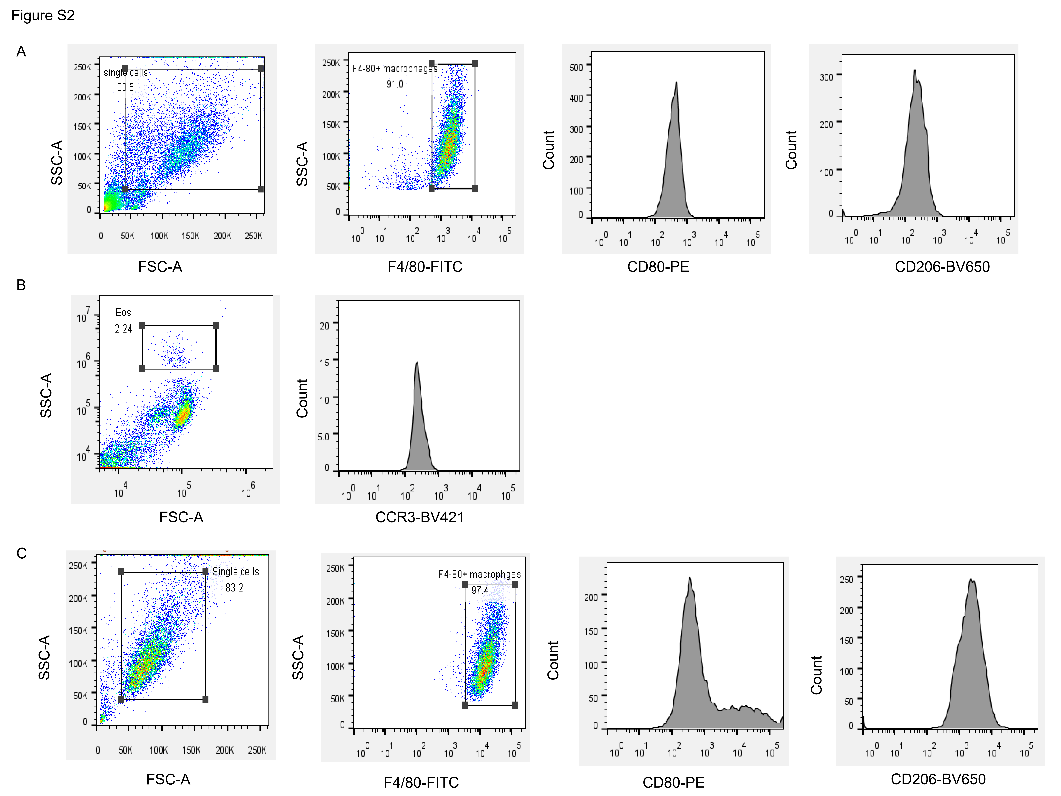


**Flow cytometry gating strategy for measuring the expression of CD80, CD206 and CCR3 from BALF and MH-S cells.** A. Expression of CD80 and CD206 in alveolar macrophages from BALF (For Figure 1F & Figure 2C). B.Expression of CCR3 in BALF (For Figure 1D & Figure 2F). C. Expression of CD80 and CD206 in IL-4-induced M2 macrophages (For Figure 3A).
