# Supplementary material for: Targeting M2 Macrophages Alleviates Airway Inflammation and Remodeling in Asthmatic Mice via miR-378a-3p/GRB2 Pathway
Source: Front Mol Biosci. 2021 Sep 13;8:717969. doi: 10.3389/fmolb.2021.717969 (PMC8473897; doi:10.3389/fmolb.2021.717969)
Supplement: Supplementary file 3 [file DataSheet3.doc]

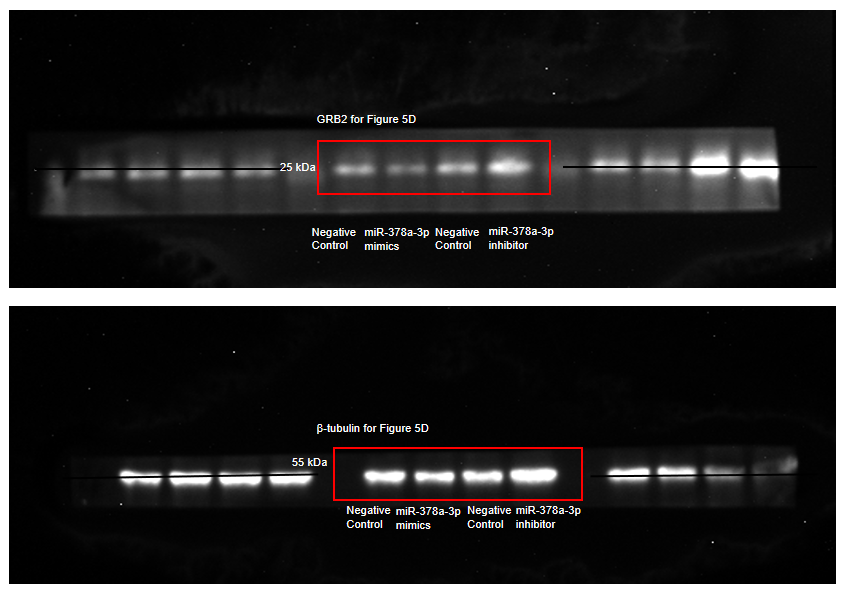


Western blotting assay of GRB2 expression in MH-S cells treated with miR-378a-3p mimics and miR-378a-3p inhibitor or control. The bands in the red box are used in the main Figure (Figure 5D).


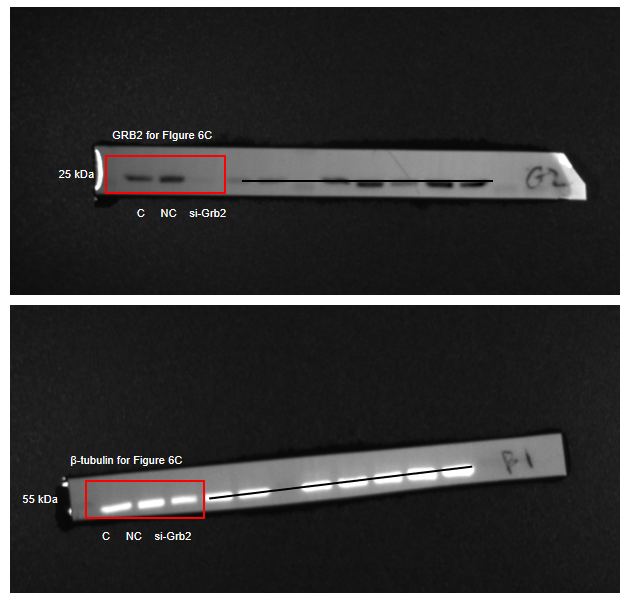


GRB2 protein in MH-S cells treated with 100 pmol si-GRB2 for 48h measured by Western Blot. The bands in the red box are used in the main Figure (Figure 6C).
